# Implementation and evaluation of the Presto combined qualitative real-time assay for *Chlamydia trachomatis* and *Neisseria gonorrhoeae* in Rwanda

**DOI:** 10.4102/ajlm.v8i1.739

**Published:** 2019-04-18

**Authors:** Vicky Cuylaerts, Irith De Baetselier, Claude M. Muvunyi, Lambert Mwambarange, Hilde Smet, John Rusine, Viateur Musengamana, Janneke van de Wijgert, Tania Crucitti

**Affiliations:** 1 Institute of Tropical Medicine, Department of Clinical Sciences, STI Reference Laboratory, Antwerp, Belgium; 2 College of Medicine and Health Sciences, University of Rwanda, Kigali, Rwanda; 3 Legacy Clinics and Diagnostics, Kigali, Rwanda; 4 National Reference Laboratory, Ministry of Health, Kigali, Rwanda; 5 Rinda Ubuzima, Kigali, Rwanda; 6 Department of Clinical Infection, Microbiology and Immunology, Institute of Infection and Global Health, University of Liverpool, Liverpool, United Kingdom

## Abstract

**Background:**

The Presto combined qualitative real-time assay for *Chlamydia trachomatis* and *Neisseria gonorrhoeae* (Presto CT/NG PCR assay) is appealing for developing countries, because it can be used with multiple DNA extraction methods and polymerase chain reaction (PCR) platforms.

**Objectives:**

The objective of the study was to implement and evaluate the Presto CT/NG PCR assay at the National Reference Laboratory (NRL) in Kigali, Rwanda, where no real-time PCR assays for the detection of *C. trachomatis* or *N. gonorrhoeae* were available.

**Methods:**

The Presto CT/NG PCR assay was first evaluated at the Institute of Tropical Medicine (ITM) in Antwerp, Belgium. Next, NRL laboratory technicians were trained to use the assay on their ABI PRISM 7500 real-time PCR instrument and their competencies were assessed prior to trial initiation. During the trial, endocervical swabs were tested at the NRL, with bi-monthly external quality control testing monitored by the ITM. The final NRL results were evaluated against extended gold standard testing at the ITM, consisting of the Abbott *m*2000 RealTi*m*e System with confirmation of positive results by an in-house real-time PCR assay for *C. trachomatis* or *N. gonorrhoeae*.

**Results:**

Of the 192 samples analysed using the Presto assay at the NRL, 16 samples tested positive for *C. trachomatis* and 17 tested positive for *N. gonorrhoeae*; four of these were infected with both. The sensitivity and specificity of the Presto assay were 93.3% (95% confidence interval [CI]: 68.1% – 99.8%) and 99.4% (95% CI: 96.8% – 100%) for *C. trachomatis* and 100% (95% CI: 76.8% – 100%) and 98.8% (95% CI: 95.8% – 99.9%) for *N. gonorrhoeae*.

**Conclusion:**

*C. trachomatis* and *N. gonorrhoeae* testing with the Presto assay was feasible in Kigali, Rwanda, and good performance was achieved.

**Keywords:**

qPCR; *Chlamydia trachomatis*; *Neisseria gonorrhoeae*.

## Introduction

*Chlamydia trachomatis* and *Neisseria gonorrhoeae* infections are the most common sexually transmitted bacterial infections worldwide.^1^ Both are frequently asymptomatic in women, which increases the risk of undiagnosed and untreated infections and of onward transmission. Untreated infections may lead to serious complications such as endometritis, salpingitis, pelvic inflammatory disease, chronic pelvic pain, ectopic pregnancy and infertility.^1,2^ In order to treat *C. trachomatis* and *N. gonorrhoeae* infections adequately, they need to be detected early and accurately.^3^

Highly sensitive and specific nucleic acid amplification assays to identify *C. trachomatis* and *N. gonorrhoeae* infections are commercially available.^4,5^ As compared to culture, the detection rates with these assays are higher. In addition, they do not require stringent sample transport conditions, because viable organisms are not necessary and they are less cumbersome compared to, for example, *Chlamydia* intracellular culture.

Over the past decades, dual detection systems for *C. trachomatis* and *N. gonorrhoeae* based on polymerase chain reaction (PCR) have been developed. Among them is the Presto combined qualitative real-time assay for *C. trachomatis* and *N. gonorrhoeae* (Presto CT/NG PCR assay; Goffin Molecular Technologies, Houten, the Netherlands). The Presto CT/NG PCR assay is characterised by its flexibility in use of sample types (with or without transport medium), DNA can be isolated using any system and it can be implemented on different real-time PCR amplification platforms.^6,7^ In addition, it provides faster results and considerably reduces the risk of contamination by amplicons compared to end-point PCR.

The assay includes an internal amplification control (IAC) to monitor both nucleic acid extraction and amplification efficiency. Clinical samples may contain inhibitory substances which are not always reliably removed during sample preparation. The specifically designed IAC will not only detect PCR inhibition but also inefficient DNA isolation from each individual sample. The IAC consists of an inactivated *Escherichia coli* containing a modified genomic DNA fragment with primer binding sites identical to the *C. trachomatis* and *N. gonorrhoeae* sequences. Detection of the amplified sequence, which is different from the *C. trachomatis* and *N. gonorrhoeae* amplicon fragments is done by a probe sequence with a different reporter dye. The Presto assay also includes a *C. trachomatis* and a *N. gonorrhoeae* selector. These selectors facilitate the detection of concurrent infections, especially when the high concentration of one infection (or target) masks the presence of the other. In other words, they are meant to select for either *C. trachomatis* or *N. gonorrhoeae* in a clinical sample with a strong positive *C. trachomatis* or *N. gonorrhoeae* result. The selector blocks the highly concentrated target present in the specimen and enhances the amplification of the other not-blocked target, if present.

Another concern that has arisen in recent years is the detection of the *C. trachomatis* Swedish variant strain. This is a *C. trachomatis* variant with a deletion in the cryptic plasmid.^8^ Amplification assays targeting the cryptic plasmid may therefore miss the detection of the *C. trachomatis* Swedish variant.^8^ According to the manufacturer, the Presto assay detects the *C. trachomatis* Swedish variant strain.

Real-time PCR assays for the detection of *C. trachomatis* and *N. gonorrhoeae* were not available at the National Reference Laboratory (NRL) in Kigali, Rwanda, in 2013. We installed the Presto assay at the NRL in the context of a clinical trial (Ring Plus study) funded by the European & Developing Countries Clinical Trials Partnership.^9,10^ We report here on the procedures followed for implementation of the Presto assay and its performance at the NRL.

## Methods

### Ethical considerations

The evaluation of the Presto assay was embedded in the clinical trial (Ring Plus study; ClinicalTrials.gov NCT01796613), which was conducted at Rinda Ubuzima Research Center, Kigali, Rwanda, from May 2013 until March 2014. The clinical trial protocol has been published elsewhere.^9^ The protocol and all study documents were reviewed and approved by the Institutional Review Board of the Institute of Tropical Medicine (ITM) (864/13), the ethics committee of the University Hospital of Antwerp (13/7/85), the Rwanda National ethics committee (122/RNEC/2014), the National Health Research Committee (NHRC/2013/PROT/0054) and the Rwandan Ministry of Health (20/2774/PHIS/ME&R/2013). All participants were between 18 and 35 years old and provided written informed consent prior to participation in the trial.

### Evaluation at the Institute of Tropical Medicine, Antwerp, Belgium

Prior to the start of the clinical trial in Rwanda, the Presto assay was evaluated at the ITM in Antwerp, Belgium.

A 1:10 dilution series of a *C. trachomatis* serovar L2 and a *N. gonorrhoeae* strain in diluted phosphate buffered saline (dPBS) was tested using the Presto assay, as well as an extended gold standard consisting of the Abbott *m*2000 RealTi*m*e System (Lake Forest, Illinois, United States) with confirmation of positive results by in-house real-time PCR assays for *C. trachomatis* and *N. gonorrhoeae*. The added value of the selectors in the Presto assay was evaluated on five aliquots containing *N. gonorrhoeae* strains in dPBS in a final concentration of 3 × 10^5^ colony forming units (CFU) per PCR reaction and five aliquots of *C. trachomatis* serovar L2 strains diluted in dPBS in a final concentration of 9 × 10^4^ elementary bodies (EB) per PCR reaction. In addition, the aliquots were spiked with a 10-fold dilution series of low concentrations of *C. trachomatis* L2 (range: 10^2^ – 10^-2^ EB per PCR reaction) and *N. gonorrhoeae* (range: 3 × 10^2^ – 3 × 10^-2^ CFU per PCR reaction). The *C. trachomatis* selector was added to the highly concentrated *N. gonorrhoeae-*positive suspensions (3 × 10^5^ CFU per PCR) and the *N. gonorrhoeae* selector was added to the highly concentrated *C. trachomatis*-positive suspensions (9 ×******* 10^4^ EB per PCR). A quality control for molecular diagnostics (QCMD) panel, consisting of 10 samples for *C. trachomatis* (QCMD 2011) and 10 samples for *N. gonorrhoeae* (QCMD 2010), was also tested with the Presto assay and the extended gold standard. Finally, the analytical specificity for *N. gonorrhoeae* was evaluated by testing eight different non-gonococcal *Neisseria* species: *N. meningitidis, N. lactamica, N. pharingis, N. animalis, N. caviae, N. ovis, N. subflava* and *N. perflava.*

### Specimen collection, processing and transport

Several sexually transmitted infection assays were performed at the clinical trial’s baseline visit,^10^ including the Presto CT/NG PCR assay on endocervical swabs (flocked swabs, Copan Technologies Srl, Brescia, Italy) that were collected by a study physician during a speculum examination.

The dry swabs were eluted in the on-site Rinda Ubuzima laboratory by adding 1.2 mL dPBS directly onto the swabs. The dPBS was prepared by dissolving 0.96 g phosphate buffered saline (PBS) and 7.65 g NaCl in 1L molecular biology water (MBW) and sterilised by filtration. After vortexing the swabs for 15 seconds, two aliquots of 550 *µ*L were prepared: one was stored at 2 °C – 8 °C until transportation in a temperature-controlled cool box with cooling elements to NRL within 1 week of sample collection (or at -20 °C in case transportation was delayed) and the other at -20 °C until transportation in a temperature-controlled dry shipper to the ITM. The Presto assay was performed at the NRL within two working days after sample receipt according to the manufacturer’s instructions.

### Training and external quality control

ITM and NRL staff wrote a standard operating procedure together, and hands-on training was conducted by ITM staff in the NRL laboratory. The competence of the trained NRL technicians was evaluated using a blind specimen panel consisting of a positive *C. trachomatis* sample, a positive *N. gonorrhoeae* sample, a sample positive for both *C. trachomatis* and *N. gonorrhoeae* and a negative sample.

External quality control panels were provided by the ITM on a bi-monthly basis throughout the study. Each panel contained a strong and a weak-positive *C. trachomatis* sample, a strong and a weak-positive *N. gonorrhoeae* sample, a sample concurrently infected with both *C. trachomatis* and *N. gonorrhoeae* and a sample negative for both *C. trachomatis* and *N. gonorrhoeae*. All samples with the exception of the weak-positive samples were expected to be correctly identified by the NRL. Weak-positive samples were allowed to be missed: they were included in the panel to encourage the NRL to excel in their competence.

### *Chlamydia trachomatis* and *Neisseria gonorrhoeae* testing using the Presto assay

The Presto assay targets the *C. trachomatis* cryptic plasmid and the *opa* gene of *N. gonorrhoeae*. Assay procedures were performed in physically separated rooms including a sample preparation room, a DNA-free area, and an amplification room. The QIAamp DNA Mini Kit (Qiagen, Hilden, Germany) was used for the DNA extraction of 400 *µ*L of aliquot, following the buccal swab spin protocol. Before extraction, 5 *µ*L of the IAC was added to every sample tube.

After adding DNA of samples and controls to the master mix, the reaction plate was loaded into the ABI PRISM 7500 instrument (Applied Biosystems™, Foster City, California, United States) with the following settings: fixed threshold, 0.01; activation polymerase, 30 sec at 95 °C; number of cycles, 40; denaturation, 3 sec at 95 °C; annealing, extension and exonuclease activity, 30 sec at 60 °C.

If a positive signal with cycle threshold (Ct) value lower than 35 was obtained for either *C. trachomatis* or *N. gonorrhoeae*, the PCR was repeated with 1 *µ*L of a selector for *N. gonorrhoeae* or *C. trachomatis* and 9 *µ*L of DNA to check for the presence of the other pathogen.

Specimens were considered negative when the Ct value of the specimen was greater than 40 and IAC was 37 or less, positive when the Ct value of the specimen was 35 or less for *C. trachomatis* or *N. gonorrhoeae* with any Ct value of the IAC, and equivocal when the Ct value of the specimen was between 35 and 40 and a Ct value of the IAC was less than 40. Specimens were considered inhibited when the Ct value of the specimen was greater than 40 and the Ct value of the IAC was greater than 37.

The assay kit contained two positive and one negative control for test-run validation which were interpreted according to the manufacturer’s instructions (CG 160501, rev05, 10/2011).

### Extended gold standard

At the ITM, DNA extraction and subsequent amplification was performed using the Abbott *m*2000 Real Ti*m*e System (Lake Forest, Illinois, United States) according to the manufacturer’s instructions. *C. trachomatis*-positive samples were confirmed with an in-house real-time PCR based on the publication by Chen et al.^11^ and *N. gonorrhoeae-*positive samples were confirmed with an in-house real-time PCR based on the publication by Hopkins et al.^12^ Confirmation of the samples was carried out on the same extracts as those used on the Abbott system and amplification was performed on the Rotor-Gene 6000 (Qiagen, Hilden, Germany).

### Statistical methods

For both *C. trachomatis* and *N. gonorrhoeae*, samples were defined as true positive if identified positive with both the Abbott assay and the in-house real-time PCR. Samples were defined as true negative when found to be negative in the Abbott assay or when a positive result could not be confirmed by the in-house real-time PCR. Prevalence at baseline was calculated for *C. trachomatis* and *N. gonorrhoeae* based on the extended gold standard. Sensitivity, specificity, positive predictive value (PPV) and negative predictive value (NPV) of the Presto assay were calculated at baseline with the extended gold standard as reference. Equivocal results in the Presto assay were considered as negative in the above mentioned calculations. Wilson Binomial 95% confidence intervals (CI) were calculated for the sensitivity, specificity, PPV and NPV.

## Results

### Evaluation at the Institute of Tropical Medicine

Evaluation of the Presto assay against the extended Gold Standard at the ITM showed that the analytical sensitivities of the Presto assay were in the same order of magnitude (100 EB/mL for *C. trachomatis*, 330 CFU/mL for *N. gonorrhoeae* versus 86 EB/mL for *C. trachomatis*, 280 CFU/mL for *N. gonorrhoeae*).

The analytical sensitivity of the Presto PCR increased by 2 log_10_ or 100 fold when a *C. trachomatis* selector was added to the strong-positive *N. gonorrhoeae* suspensions. The Ct value of the IAC decreased, reducing the inhibition. No difference in sensitivity was obtained by using a *N. gonorrhoeae* selector on strong-positive *C. trachomatis* suspensions, although the Ct value of the IAC also decreased. No effect of the use of selectors was obtained when strong *C. trachomatis*-positive clinical samples were spiked with low concentrations of *N. gonorrhoeae* or when strong *N. gonorrhoeae-*positive clinical samples were spiked with low concentrations of *C. trachomatis*.

The QCMD panel included eight positive and two negative *C. trachomatis* samples. Six samples tested *C. trachomatis-*positive by the Presto assay. Of the two low-positive *C. trachomatis* samples that were not detected by the Presto assay, one was also missed using the extended gold standard. The QCMD panel included a *C. trachomatis* Swedish variant, which was successfully detected with the Presto assay. The QCMD panel for *N. gonorrhoeae* included seven positive and three negative *N. gonorrhoeae* samples, of which one was *Neisseria cinerea.* Six samples tested positive in the Presto assay. One sample was not tested due to insufficient volume to perform the test. The three *N. gonorrhoeae-*negative samples tested negative in the Presto assay.

No cross reactivity was detected with the other eight *Neisseria* species.

### Implementation of the Presto assay at the National Reference Laboratory

The results of the blind specimen panel used for competency check were 100% concordant. The bi-monthly external quality control panels also produced satisfactory results: two *C. trachomatis* weak-positive samples were missed with the Presto assay, and one *N. gonorrhoeae-*positive sample result was equivocal.

### Clinical trial results

Baseline samples from 185 women were tested for *C. trachomatis* and *N. gonorrhoeae* by the Presto assay. Additional samples from seven women were tested at follow-up visits. The results are summarised in Table 1.

**TABLE 1:** Presto CT/NG PCR assay results, National Reference Laboratory, Kigali, Rwanda, May 2013 -March 2014.

Infection	Presto				
	Positive	Negative	Equivocal^†^	Inhibition^‡^	Total
CT	16^§,‡‡^	167	8^¶^	1	192
NG	17^§,§§^	166	8^††^	1	192

CT, *Chlamydia trachomatis*; NG, *Neisseria gonorrhoeae*.

† , Equivocal: when the cycle threshold (Ct) value of the specimen is between 35 and 40 and that of the isolation amplification control (IAC) lower than 40.

‡ , Inhibited specimen: when the Ct value of the specimen was > 40 and of the IAC > 37.

§ , Four samples with concurrent infections of which one was symptomatic: burning while urinating and abnormal discharge.

¶ , Two symptomatic: abnormal unusual vaginal discharge and enlarged inguinal lymph nodes.

†† , Two symptomatic: abnormal unusual vaginal discharge.

‡‡ , Two symptomatic: one burning when urinating, one abnormal unusual vaginal discharge.

§§ , Two symptomatic: one burning when urinating, one abnormal unusual vaginal discharge.

All study samples (*n* = 192) were retested according to the extended gold standard algorithm at the ITM. Two samples were excluded from data analysis as they contained PCR inhibitors: one sample was inhibited in the Presto assay and one sample was inhibited in the Abbott assay. The Presto assay missed one *C. trachomatis-*positive sample, although this was equivocal in the assay. It falsely detected three *N. gonorrhoeae* and one *C. trachomatis*. The latter *C. trachomatis* sample was positive on the Abbott assay but could not be confirmed using the in-house real-time PCR (Table 2).

**TABLE 2:** Cross-tabulation of the Presto CT/NG PCR assay and extended gold standard *Chlamydia trachomatis* and *Neisseria gonorrhoeae* results for *Chlamydia trachomatis* National Reference Laboratory, Kigali, Rwanda, May 2013 -March 2014.

Presto (CT)	Extended gold standard (CT)			Presto (NG)	Extended gold standard (NG)		
	Positive	Negative	Total		Positive	Negative	Total
Positive	15	1^‡^	16	Positive	14	3	17
Negative	1^†^	173	174	Negative	0	173	173
Total	16	174	190	Total	14	176	190

Note: Presto equivocal results were considered as negative. Values in bold signify discordant results.

CT, *Chlamydia trachomatis*; NG, *Neisseria gonorrhoeae*.

† , Equivocal in Presto;

‡ , Positive in Abbott assay but not confirmed by in-house real-time PCR.

At baseline, the overall prevalence was 8.1% for *C. trachomatis* and 7.0% for *N. gonorrhoeae*. The sensitivity, specificity, PPV and NPV of the Presto assay are summarised in Table 3.

**TABLE 3:** Diagnostic characteristics of the Presto assay National Reference Laboratory, Kigali, Rwanda, May 2013 – March 2014.

Infection	Sensitivity (95% CI)	Specificity (95% CI)	PPV (95% CI)	NPV (95% CI)
CT	93.3% (68.1–99.8)	99.4% (96.8–100)	93.3% (66.4–99.0)	99.4% (96.2–99.9)
NG	100% (76.8–100)	98.8% (95.8–99.9)	86.7% (62.1–96.3)	100%

Note: Presto equivocal results were considered as negative. Diagnostic characteristics based on samples collected at baseline.

CI, confidence interval; CT, *Chlamydia trachomatis*; NG, *Neisseria gonorrhoeae*; NPV, negative predictive value; PPV, positive predictive value.

## Discussion

We successfully implemented the Presto combined qualitative real-time assay for *Chlamydia trachomatis* and *Neisseria gonorrhoeae* at the NRL, Kigali, Rwanda. The implementation was a stepwise process covering hands-on training and writing of standard operating procedures. The competency of the laboratory technicians in the execution of the Presto assay was confirmed through the 100% concordant results obtained for the testing of the blind specimen panel. The testing of the bi-monthly external quality control panels allowed us to monitor and advise the NRL on testing for *C. trachomatis* and *N. gonorrhoeae* using the Presto assay. In the end, we were able to successfully install the Presto assay at the NRL and to guarantee the quality of the results during the clinical trial. In addition, the quality control procedures and retesting of samples at ITM may contribute to the method validation that is required for laboratory accreditation. The NRL may also use a similar implementation approach in the event the amplification testing for *C. trachomatis* and *N. gonorrhoeae* should be used in other settings such as for diagnosis or for research or clinical trials.

The analytical sensitivity of the Presto assay for *C. trachomatis* and *N. gonorrhoeae* was comparable to that of the extended gold standard. Previous versions of PCR assays for *N. gonorrhoeae* showed cross reactivity and amplification of non-gonorrhoeae *Neisseria* species,^13^ which increased the ratio of false-positive *N. gonorrhoeae* detection and resulted in unnecessary treatment. Superfluous antibiotic use increases the risk of untreatable *N. gonorrhoeae* due to multidrug resistance and should be maximally prevented.^14^ In the brief evaluation study of the Presto assay at the ITM, none of the non-gonorrhoeae *Neisseria* species was amplified by the Presto assay, confirming its analytical specificity. However, the PPV for *N. gonorrhoeae* detection using the Presto assay was under 90%, which necessitates a confirmation test for *N. gonorrhoeae* as recommended by the International Union against Sexually Transmitted Infections (IUSTI) guidelines.^15^ PPVs are influenced by prevalence and specificity of the assay, and in this study the lower PPV was mainly caused by two *N. gonorrhoeae* false-positive samples.

The testing for *C. trachomatis* and *N. gonorrhoeae* described in this manuscript was part of a clinical trial which was conducted according to Good Clinical and Laboratory Practice requirements.^9^ The delivered results were quality assured, and the traceability and transparency of the test procedures and handlings within the laboratory were guaranteed. The obtained and reported sensitivity and specificity reflect the potentially achievable performance of the assay in a quality-assured environment within the NRL in Kigali, Rwanda.

Our study population was characterised by a high prevalence of both *C. trachomatis* and *N. gonorrhoeae*. Almost one quarter of the infections were dual infections, highlighting the need for a dual detection system in this setting. The advantages of a dual detection system are staff time and assay cost reduction while two detection results are delivered. Notwithstanding this study’s small sample size, the Presto assay’s sensitivity and specificity for *C. trachomatis* detection was in line with two previous studies.^6,7^ We cannot explain the lower specificity found for *N. gonorrhoeae* detection; we did not obtain cross reactions with the non-gonorrhoeae *Neisseria* species, and we therefore advise that a possible lower specificity should be confirmed in future studies. A total of eight equivocal results (4.2%) were found during this small evaluation. In clinical practice, clinicians should be instructed to collect another sample for retesting. The result will be reported as equivocal in the event of a second equivocal result and conclusion regarding the diagnosis cannot be drawn, or as positive or negative, if the second test result is positive or negative.

To date no reports have been found on the current presence of the *C. trachomatis* Swedish variant strain in Rwanda or Africa, but it has become a global requirement that newly designed PCR assays for *C. trachomatis* detection also detect the *C. trachomatis* Swedish variant.^8^ Although the *C. trachomatis* target of the Presto assay lies within the *C. trachomatis* cryptic plasmid, the manufacturer states that the *C. trachomatis* Swedish variant will be detected. This statement was confirmed by testing a QCMD panel which included a sample with the *C. trachomatis* Swedish variant.

The laboratory and etiological diagnosis of *C. trachomatis* and *N. gonorrhoeae* is scarcely available and problematic in resource-poor settings. To date many settings have adopted the syndrome-based management approach.^16^ However, limitations of the vaginal discharge algorithm, and in particular the management of gonococcal and chlamydia infections, has been recognised.^17,18^ The approach guides the diagnosis of *C. trachomatis* and *N. gonorrhoeae* based on symptoms and signs.^14,19^ Per definition, asymptomatic infections are not recognised and not treated; on the other hand, symptomatic treatment increases the risk of overtreatment.^20^ Although attempts have been made to develop rapid diagnostic assays for the detection of *C. trachomatis* and *N. gonorrhoeae*, none, except one point-of-care test, the Cepheid GeneXpert CT/NG assay, is sufficiently accurate to be useful in an etiological diagnosis.^21,22^ The results of our study and feedback from the NRL staff suggest that the Presto assay can be used in a molecular laboratory within a resource-limited context. The performance of the assay is comparable to other commercial amplification assays and has the advantage that it can be run on any amplification platform available in standard molecular laboratories, thus avoiding extra investment costs. At the time of this evaluation, we paid €17.00 per sample including all consumables and reagents. However, the manufacturer recently revised the Presto kit prices; the current cost per sample should be around €10 inclusive of consumables and reagents. The GeneXpert is the only molecular assay for *C. trachomatis* and *N. gonorrhoeae* that may outcompete the Presto assay in developing country settings. It is faster, but not as flexible, and it requires its own GeneXpert equipment, which is widely used in developing countries’ laboratories for tuberculosis diagnostic testing. Still, the reagent for the GeneXpert CT/NG assay is more expensive compared to the Presto assay.

### Limitations

An initial evaluation of the Presto assay was done in Belgium at the ITM. Ideally, this should have been performed in Rwanda, the country of use. However, at that time a PCR assay for the detection of *C. trachomatis* and *N. gonorrhoeae* was not applied at the NRL in Kigali, and well-characterised reference, clinical or quality control panels were not available. We acknowledge that the inability to initially evaluate the Presto assay in Rwanda is a limitation of the study.

### Conclusions

In conclusion, testing for *C. trachomatis* and *N. gonorrhoeae* with the Presto assay was easily implemented and feasible in Kigali, Rwanda. Overall, a good performance of the assay was achieved, but our results suggest that positive Presto *N. gonorrhoeae* results may need confirmation using a nucleic acid amplification assay with a different amplification target. Further field evaluations are recommended to confirm our findings.
